# Timosaponin AIII promotes non-small-cell lung cancer ferroptosis through targeting and facilitating HSP90 mediated GPX4 ubiquitination and degradation

**DOI:** 10.7150/ijbs.77979

**Published:** 2023-02-27

**Authors:** Cong Zhou, Ting Yu, Rui Zhu, Junjie Lu, Xiaohu Ouyang, Zili Zhang, Qianyun Chen, Junyi Li, Jing Cui, Feng Jiang, Kim Yun Jin, Alexey Sarapultsev, Fangfei Li, Ge Zhang, Shanshan Luo, Desheng Hu

**Affiliations:** 1 Department of Integrated Traditional Chinese and Western Medicine, Union Hospital, Tongji Medical College, Huazhong University of Science and Technology, Wuhan, China.; 2 Health Management Center, Hubei Provincial Hospital of Integrated Chinese & Western Medicine, Wuhan, China.; 3 College of International Education, Tianjin University of Traditional Chinese Medicine, Tianjin, China.; 4 School of Traditional Chinese Medicine, Xiamen University Malaysia, Selangor. 43900. Malaysia.; 5 Russian-Chinese Education and Research Center of System Pathology, South Ural State University, 454080 Chelyabinsk, Russia.; 6 Institute of Immunology and Physiology, Ural Branch of the Russian Academy of Science, 620049 Ekaterinburg, Russia.; 7 Shum Yiu Foon Sum Bik Chuen Memorial Centre for Cancer and Inflammation Research, School of Chinese Medicine, Hong Kong Baptist University, 999077, Hong Kong SAR, China.; 8 Institute of Integrated Bioinfomedicine and Translational Science, School of Chinese Medicine, Hong Kong Baptist University, Hong Kong SAR, China.; 9 Institute of Hematology, Union Hospital, Tongji Medical College, Huazhong University of Science and Technology, Wuhan, China.; 10 Key Laboratory of Biological Targeted Therapy, the Ministry of Education, Wuhan, China.; 11 Clinical Research Center of Cancer Immunotherapy, Wuhan, China.

**Keywords:** Timosaponin AIII, heat shock protein 90, glutathione peroxidase 4, ferroptosis, non-small cell lung cancer

## Abstract

Timosaponin AIII (Tim-AIII), a steroid saponin, exhibits strong anticancer activity in a variety of cancers, especially breast cancer and liver cancer. However, the underlying mechanism of the effects of Tim-AIII-mediated anti-lung cancer effects remain obscure. In this study, we showed that Tim-AIII suppressed cell proliferation and migration, induced G2/M phase arrest and ultimately triggered cell death of non-small cell lung cancer (NSCLC) cell lines accompanied by the release of reactive oxygen species (ROS) and iron accumulation, malondialdehyde (MDA) production, and glutathione (GSH) depletion. Interestingly, we found that Tim-AIII-mediated cell death was reversed by ferroptosis inhibitor ferrostatin-1 (Fer-1). Meanwhile, the heat shock protein 90 (HSP90) was predicted and verified as the direct binding target of Tim-AIII by SwissTargetPrediction (STP) and surface plasmon resonance (SPR) assay. Further study showed that Tim-AIII promoted HSP90 expression and Tim-AIII induced cell death was blocked by the HSP90 inhibitor tanespimycin, indicating that HSP90 was the main target of Tim-AIII to further trigger intracellular events. Mechanical analysis revealed that the Tim-AIII-HSP90 complex further targeted and degraded glutathione peroxidase 4 (GPX4), and promoted the ubiquitination of GPX4, as shown by an immunoprecipitation, degradation and* in vitro* ubiquitination assay. In addition, Tim-AIII inhibited cell proliferation, induced cell death, led to ROS and iron accumulation, MDA production, GSH depletion, as well as GPX4 ubiquitination and degradation, were markedly abrogated when HSP90 was knockdown by HSP90-shRNA transfection. Importantly, Tim-AIII also showed a strong capacity of preventing tumor growth by promoting ferroptosis in a subcutaneous xenograft tumor model, whether C57BL/6J or BALB/c-nu/nu nude mice. Together, HSP90 was identified as a new target of Tim-AIII. Tim-AIII, by binding and forming a complex with HSP90, further targeted and degraded GPX4, ultimately induced ferroptosis in NSCLC. These findings provided solid evidence that Tim-AIII can serve as a potential candidate for NSCLC treatment.

## Introduction

For several decades, lung cancer has been one of the most common cancers and the leading cause of cancer-related deaths worldwide 1. According to histological classification, around 87% of lung cancers are non-small cell lung cancer (NSCLC) 2. The main therapies for NSCLC are surgery, radiation therapy, chemotherapy, and immunotherapy 3. Surgery is the best option for patients with NSCLC in the early-stage. However, quite a number of patients with NSCLC were first diagnosed at already inoperable stage or experienced relapses of the disease after surgery. Chemotherapy and radiotherapy are the basic treatments for solid tumors 4, 5. Although there has been great progress in immunotherapy, the efficacy is still unsatisfactory 6. Therefore, finding new antitumor components besides current therapies for patients with NSCLC will be essential to improve the survival rate of patients.

Induction of tumor cell death is an emerging approach for cancer therapy. Some modalities of programmed cell death, such as autophagy 7, 8, apoptosis 9, and necrosis 10 play an important role in tumor treatment. Ferroptosis, an iron-dependent form of cell death, is generally considered a type of ROS-dependent cell death 11. Evidence has shown that induction of ferroptosis is considered to be a novel anticancer strategy. RSL3, a ferroptosis inducer, inhibits tumor growth in a BJeLR cell-derived xenograft mouse model 12. Erastin (a ferroptosis inducer) can be combined with various drugs such as cisplatin, temozolomide, and doxorubicin for the anticancer treatment of different types of cancers 13. Furthermore, a study of Wan C et al (2020) revealed a potential relationship between ferroptosis and radiation-induced bystander effect (RIBE), demonstrating that irradiated tumor cell-released microparticles are the primary mediators of RIBE, leading to broad antitumor effects via ferroptosis 14. These findings indicated that ferroptosis induction can be utilized as an interventional target for tumor treatment.

It is well known that intracellular reactive oxygen species (ROS) and iron accumulation, lipid peroxidation, and glutathione (GSH) depletion are crucial processes during ferroptosis 15. Malondialdehyde (MDA) is an indirect indicator of ROS-induced lipid peroxidation-induced injury. Glutathione peroxidase 4 (GPX4), a glutathione peroxidase, plays an essential role in inhibiting ferroptosis 16. GPX4 specifically catalyzes the oxidative activity of lipid peroxides in a glutathione-dependent manner, thus protecting cells from the threat of ferroptosis 12, 17, 18. GPX4 knockdown induces renal cell carcinoma ferroptosis accompanied by ROS accumulation 12. ROS produced by extracellular or intracellular stimuli play a fundamental role in cell and tissue damage in a variety of diseases 19, 20. It has recently been reported that ferroptosis is a natural tumor suppression process, and inactivation of ferroptosis contributes to tumor development of tumor 16, 21-24.

The natural product *Anemarrhena Asphodeloides* Bunge has a long history of being used to treat arthralgia, hematochezia, tidal fever, and night sweats in traditional Chinese medicine 25. Tim-AIII, a steroidal saponin, is the main active ingredient of* Anemarrhena Asphodeloides* Bunge, which exhibits multiple pharmacological activities, such as anti-cancer 26-28, anti- neurodegenerative 29, anti-inflammatory 30, anti-coagulant 31, etc. Tim-AIII mediated effects on cancers, especially in liver cancer 27, 32 and breast cancer 26, 33 are supposed as its most potential activity. However, the role, target, and the underlying molecular mechanism in lung cancer are still poorly understood.

In this study, we investigated the effects of Tim-AIII in NSCLC cells and further explored the underlying mechanisms *in vitro* and* in vivo*. Our data showed that Tim-AIII induced cell death and G2/M arrest and inhibited the migration of NSCLC cells. Further studies demonstrated that Tim-AIII has therapeutic effect on NSCLC by binding to its target HSP90, which promotes ubiquitination and degradation of GPX4 ultimately induces NSCLC cells ferroptosis. These findings highlight the role of Tim-AIII in the treatment of NSCLC and reveal a potential natural drug for NSCLC therapy.

## Materials and Methods

### Materials

Tim-AIII (purity >98%) was purchased from Chengdu Herbpurify Co., LTD (Z-019, China) and was dissolved in dimethyl sulfoxide (DMSO, 196055, MP Biomedicals, USA) to prepare a stock solution of 10 mM and stored at -20°C. Mouse anti-GPX4 (67763-1-Ig) antibody, rabbit anti-HSP90 (13171-1-AP), E-Cadherin (20874-1-AP), Vimentin (10366-1-AP), Snail-1 (13099-1-AP), Snail-2 (12129-1-AP), N-Cadherin (22018-1-AP) antibodies and HRP-conjugated anti-heavy chain of rabbit IgG antibody (SA00001-7H) were purchased from Proteintech (Wuhan, China). Rabbit anti-GPX4 (A1933), FTL (A11241), HMOX-1 (A1346), SLC40A1 (A14885), SLC7A11 (A13685) Ubiquitin (A19686) and β-actin (AC004) antibodies were purchased from ABclonal (Wuhan, China). The HRP-labeled goat anti-rabbit IgG and HRP-labeled goat anti-mouse secondary antibody was purchased from Beyotime (Shanghai, China). Chloroquine (CQ, HY-17589A), Z-VAD-FMK (HY-16658B), Necrostatin-1 (Nec-1, HY-15760), Ferrostatin-1 (Fer-1, HY-100579), N-acetylcystein (NAC, HY-B0215), tanespimycin (HY-10211), trolox (HY-101445), cycloheximide (CHX, HY-12320) and MG-132 (HY-13259) were purchased from Med Chem Express (Shanghai, China). The recombinant human HSP90 alpha protein (ab48801) was provided by Abcam (Shanghai, China). The recombinant Human GPX4 protein (P0633) was provided by Fine Biotech (Wuhan, China).

### Cell lines and cell culture

Human NSCLC cells (H1299, A549, SPC-A1), mouse NSCLC cells (Lewis lung carcinoma, LLC) and human bronchial epithelial (HBE) cells were gifts from the Respiratory Laboratory of Wuhan Union Hospital (China). LLC cells were cultured in Dulbecco's modified Eagle's medium (DMEM) (Gibco, United States) containing 10% fetal bovine serum (FBS) (Gibco, United States) and A549 cells were cultured in DME/F-12 (Hyclone, United States) containing 10% FBS. H1299, SPC-A1 and HBE cells were cultured at Roswell Park Memorial Institute (RPMI)-1640 (Gibco, United States) containing 10% FBS. Cells were incubated at 37 ° C in a humidified atmosphere with 5% CO_2_. Cells in logarithmic growth phase were collected for further experiments, when H1299, A549, SPC-A1 and LLC cells covered 90% of the bottom of the culture plate.

### In *vivo* tumor model

Five-week-old male C57BL/6J mice and BALB/c-nu/nu nude mice were purchased from Beijing Vital River Laboratory Animal Technology Co., Ltd. (Beijing, China). All animals were housed in a specific pathogen-free animal facility and kept in a temperature and humidity- controlled environment (25 ± 2 °C, 50 ± 5% humidity) with free access to food and water on a standard 12h light/12h dark cycle. The protocols and the use of animals have been approved by the Animal Experiment Center of Huazhong University of Science and Technology (SYXK-2021-0057). 100 μL media containing 8×10^5^ LLC cells was injected subcutaneously into the right flanks of male C57BL/6J mice and 100 μL media containing 1.6×10^6^ H1299 cells was injected subcutaneously into the right flanks of male BALB/c-nu/nu nude mice. After 7 days, the mice were randomly grouped into three groups, eight mice per group, and Tim-AIII was injected intraperitoneally every other day and the body weight and tumor volume of the mice were recorded after injection. Tumor volume was evaluated every two days and was calculated using the following formula: (short diameter)^2^ × (long diameter)/2. 100 μL media containing 8×10^5^ LLC cells were injected into C57BL/6J mice through the tail vein to establish a tumor model of lung metastasis. After 7 days, the mice were grouped randomly into three groups, eight mice per group. After 26 days of Tim-AIII administration, the mice were sacrificed and lung tissues were collected.

### Analysis of Cell Proliferation and cytotoxicity

Cell proliferation was evaluated using a cell counting kit-8 assay (CCK-8, GK10001, GlpBio, USA). Briefly, H1299, A549, SPC-A1 and LLC cells were treated with DMSO or Tim-AIII for 24 h, 48 h and 72 h. After treatment, the CCK-8 reagent was added and cells were further incubated at 37 ° C for 1 h and absorbance was measured at 450 nm on a microplate reader (Thermo Scientific). Cell cytotoxicity was detected using a lactate dehydrogenase (LDH) cytotoxicity assay kit (C0017, Beyotime, China). Briefly, H1299 and A549 cells were treated with DMSO or Tim-AIII for 24 h, 48 h and 72 h. Following treatment, the cell supernatant was collected and the LDH detection reagent was added. The cells were further incubated on a shaker at room temperature (RT) for 30 min and absorbance was measured at 490 nm on a microplate reader (Thermo Scientific).

### Colony Formation Assays

For colony formation assays, H1299 and A549 cells were cultured in a 10 cm dish with 4×10^3^ cells for 1 week. After washing twice with phosphate buffer saline (PBS), the cells were fixed with 4% paraformaldehyde for 15 min at RT and then stained with 0.1% crystal violet (G1014, Servicrbio, China) for 30 min. The numbers of colonies were counted for analysis.

### Analysis of the cell cycle

A propidium iodide (PI) cell cycle staining Kit (70-CCS012, Multi Sciences, China) was utilized to detect the cell cycle by flow cytometry. This determination was based on the measurement of the DNA content of nuclei labeled with PI. Cells were prepared by trypsinization and washed twice with PBS, then stained with 1 ml of DNA staining solution and 10 μL Permeabilization solution. Flow cytometry was performed on Beckman Coulter CytoFLEX and data were analyzed with CytExpert 2.4 software.

### Wound healing and migration assays

For wound healing assays, the cells were scratched with a sterile plastic tip when they covered the bottom of the 6-well plate, and then cultured in a serum-free medium supplemented with Tim-AIII for 24 h, then observed and photographed with an optical microscope (Olympus, CKX53, Japan).

The migration assay was performed in a 24-well cell culture chamber inserted with 8 μm pores (Corning, USA). The inserts containing 4×10^3^ cells in the upper chamber, and cultured with Tim-AIII for 24 h. After culture, the cells on the upper surface were removed. The cells on the reverse side were fixed with 4% paraformaldehyde for 15 min and then stained with crystal violet. Finally, the migrated cells were observed and photographed under an optical microscope at 200× magnification.

### Measurement of lipid ROS

The lipid ROS levels were detected by lipid peroxidation senso (BODIPY™ 581/591 C11, D3861, Invitrogen, USA).H1299 and A549 cells were cultured in 6-well plates with a density of 2 × 10^5^ cells per well. After incubation with Tim-AIII for 48 h, cells were gently washed with PBS and followed by incubation with BODIPY™ 581/591 C11 (10 μM) for 30 min at 37 °C. The level of lipid ROS was measured by a BECKMAN COULTER flow cytometer.

### Measurement of irons

The iron colorimetric assay kit (E-BC-K880-M, Elabscience, Wuhan) was used to detect total intracellular irons. Briefly, H1299 and A549 cells were cultured in 6-well plates with a density of 2 × 10^5^ cells per well. Following incubation with Tim-AIII for 48 h, cells were harvested and lysed. The supernatant was collected by centrifugation to detect the level of irons.

### MDA assay

Total MDA were measured using an MDA assay kit (TBA method, A003-1-2) purchased from Jiancheng (Nanjing, China). Briefly, H1299 and A549 cells were cultured in 6-well plates with a density of 2 × 10^5^ cells per well. Following incubation with Tim-AIII for 48 h, cells were harvested, then resuspended in PBS and lysed by sonication. The supernatant was collected by centrifugation to detect the level of MDA.

### GSH assay

Total GSH were measured using a GSH assay kit (A006-2-1) purchased from Jiancheng (Nanjing, China). Briefly, H1299 and A549 cells were cultured in 6-well plates with a density of 2 × 10^5^ cells per well. Following incubation with Tim-AIII for 48 h, cells were harvested, then resuspended in PBS and lysed by sonication. The supernatant was collected by centrifugation to detect the level of GSH.

### Mitochondrial membrane potential (MMP) assay

Mitochondrial membrane potentials of H1299 and A549 cells were measured using the fluorescent probe JC-1 (Beyotime, C2005). After treatment, cells were gently stained with JC-1 for 20 min at 37 ° C. Also, the fluorescent intensity of JC-1 monomers (FITC channel) and JC-1 aggregates (PE channel) was quantified by a BECKMAN COULTER flow cytometer.

### Transmission electron microscope (TEM)

The cultured cells were fixed with a solution containing 2.5% glutaraldehyde in PBS for 24 h in a 10 cm plate. After washing with PBS, cells were treated with 0.1% Millipore-filtered cacodylate buffered tannic acid, postfixed with 1% buffered osmium, and stained with 1% Millipore-filtered uranyl acetate. After dehydration and embedding, the samples were incubated in a 60 ° C oven for 24 h. Digital images were obtained using a TME.

### Surface Plasmonic Resonance (SPR) Assay

Install the COOH chip according to the standard operating procedure of the Open SPRTM instrument. Dilute the analyte with 1% DMSO and loaded at 20 μL/min. The binding time of protein and ligand was 240 s; the natural dissociation was 300 s. The One-to-One analysis model with Trace Drawer software (Ridgeview Instruments ab, Sweden) was used for the analysis.

### Generation and purification of the HSP90 mutant protein

The mutant protein was purified by using BeyoGold TM His tag purification resin (denaturant resistant) (P2233, Beyotime Biotechnology, Shanghai, China) according to the manufacturer's protocol. Briefly, the coding sequence of the human mutant HSP90 gene (amino acids at sites 51, 58, and 138 were mutated to alanine) with the His tag was cloned into a vector tagged pET28A and transformed into E. *coli* BL21 star (DE3) cells. Isopropyl β-D-thiogalactoside (IPTG) was added to induce the expression of the HSP90-His protein, and E. *coli* were harvested after incubation at RT for 6 h. Total protein in E. *coli* is obtained by lysing solution and lysozyme. The histidine residues on the His tag of the recombinant protein could specifically bind to the nickel ions in BeyoGold™ His-tag Purification Resin (denaturation-resistant formulation). After washing, the His-tag recombinant protein was eluted under native or denaturing conditions for separation and purification.

### Western blot (WB) analysis

Cells and subcutaneous xenograft tumor samples were lysed with RIPA lysate (G2002, Servicebio, Wuhan, China) to extract total protein after treatment with Tim-AIII. The protein samples were loaded onto 4% or 10% or 12% sodium dodecyl sulfate-polyacrylamidegel electrophoresis gels (SDS-PAGE) gels and then transferred to a 0.45 μm PVDF membrane. PVDF membrane was immersed in TBST containing 5% skim milk and blocked at RT on a shaker for 1.5 h, then incubated with specific primary antibodies on a shaker overnight at 4 ° C. The PVDF membrane was immersed in the secondary antibody incubation solution for 1.5 h after being rinsed with TBST three times, 10 min for each time. After incubation with the ELC reagent (a mixture of solution A and B in equal proportion), the membranes were visualized by imaging system (iBright 750, Invitrogen, USA).

### Quantitative real-time PCR analysis

The cells were first cultured in the presence of different concentrations of Tim-AIII for 48 h. Cell pellets were treated with RNAiso plus (9109, TaKaRa, Japan) reagent to obtain total RNA. Total RNA was reversely transcribed into cDNA using HiScript III RT SuperMix (R323-01, Vazyme, Nanjing). Then real-time PCR was performed on an ABI 7300 Real-time PCR instrument using the AceQ qPCR SYBR Green MasterMix (Q111-02/03, Vazyme, Nanjing) system: 95 ° C for 15 min, 95 °C for 10 s, and 60 °C for 30 s, 40 cycles. Finally, a dissolution curve was drawn and the 2^^(-△△CT)^ method was used to analyze the final data. GAPDH and β-actin was used as internal reference. The gene primer sequences were obtained from NCBI (Bethesda, MD, USA). The primer sequences are shown in** Table 1**.

### Co-immunoprecipitation (Co-IP)

H1299 and A549 cells were seeded in 10 cm culture plates overnight and incubated with 4 μM Tim-AIII or the same amount of DMSO. After incubation, cells were harvested and treated with IP lysis solution (P0013J, Beyotime, Shanghai, China) to obtain proteins. The cell lysates were incubated with the HSP90 antibody with 1:100 dilution overnight at 4 ° C. After further incubation with protein A/G Sepharose beads (P2179, Beyotime, Shanghai, China) for 3 h at 4 °C, the beads were washed five times with IP lysis solution and added loading buffer for WB.

### In vitro ubiquitination assay

H1299 and A549 cells were seeded in 6 cm culture dishes overnight and incubated with 4 μM Tim-AIII or equal amount of DMSO for 48 h. Then, 10 μM MG-132 was added to inhibit the protein degradation for 4 h. After incubation, the cells were harvested and treated with IP lysis solution (P0013J, Beyotime, Shanghai, China) to obtain proteins. Then, the cell lysates were incubated with the GPX4 antibody with 1:100 dilution overnight at 4 °C. Following further incubation with protein A/G Sepharose beads (P2179, Beyotime, Shanghai, China) for 3 h at 4 °C, the beads were washed five times with IP lysis solution and adding loading buffer for WB.

### Degradation of the GPX4 assay

The recombinant human HSP90 was incubated in PBS with recombinant human GPX4 in the presence or absence of a different amount of Tim-AIII for 1 h at 37 ° C with shaking. Next, we add loading buffer for WB analysis.

### Generation of stable cell lines

To generate a stable HSP90 knockdown cell line, the lentiviral vector pLenti-GV112 puromycin (GeneChem, Shanghai, China) was constructed and used for the corresponding cells by lentivirus-mediated transfection. The target sequence of HSP90 shRNA (GCAGCCATTTATATTGCTTAG) and the corresponding control shRNA (TTCTCCGAACGTGTCACGT) were designed by GeneChem (Shanghai, China). H1299 and A549 cells in the logarithmic growth phase were transfected by adding 20 μL 1 × 10^8^ TU/mL lentivirus in a six-well dish with 2 × 10^5^ cells per well. After 72 h of transfection, transfection efficiency and knockout efficiency were assessed by fluorescence microscopy and WB.

### Biochemical analysis

The serum alanine aminotransferase (ALT), aspartate aminotransferase (AST), creatinine (Cre), and blood urea nitrogen (BUN) content were detected by ALT assay kit (C009-2-1, Jiancheng, Nanjing, China), the AST assay kit (C010-2-1, Jiancheng, Nanjing, China), the urea assay kit (C013-2-1, Jiancheng, Nanjing, China) and the creatinine assay kit (C011-2-1, Jiancheng, Nanjing, China), respectively.

### Flow cytometry analysis

Single cell suspensions were obtained as previously described 34. Cells were stained for live/dead with FVD, followed by staining with antibodies specific for CD45, CD3, CD19, CD4, CD8, IFN-γ, Foxp3, NK1.1, F4/80, CD11c, CD206, as well as CD11b. For IFN-γ and Foxp3 staining, cells were first cultured with phorbol 12-myristate 13-acetate (PMA, sc-3576, Santa Cruz Biotechnology), ionomycin (ALX-450-007, ALEXIS Biochemicals) and brefeldin A (423303, BioLegend) for 6 h, and followed by intracellular staining using an intracellular staining kit (554714, BD Biosciences). Flow cytometry was performed on Beckman Coulter CytoFLEX and data were analyzed with CytExpert 2.4 software.

### Statistical Analysis

All data were presented as mean ± standard deviation (SD), analyzed using SPSS version 23.0 software and graphed using GraphPad Prism version 8 software. Differences between groups were analyzed by one-way ANOVA or Student's t test. *P* values less than 0.05 were considered to indicate significance. and *p* values were labeled as follows: ^*^p< 0.05; ^**^p< 0.01; ^***^p< 0.001.

## Results

### Tim-AIII triggers cell death, inhibits cell proliferation, and promotes cell cycle arrest in G2/M in NSCLC cells

**Figure 1:**
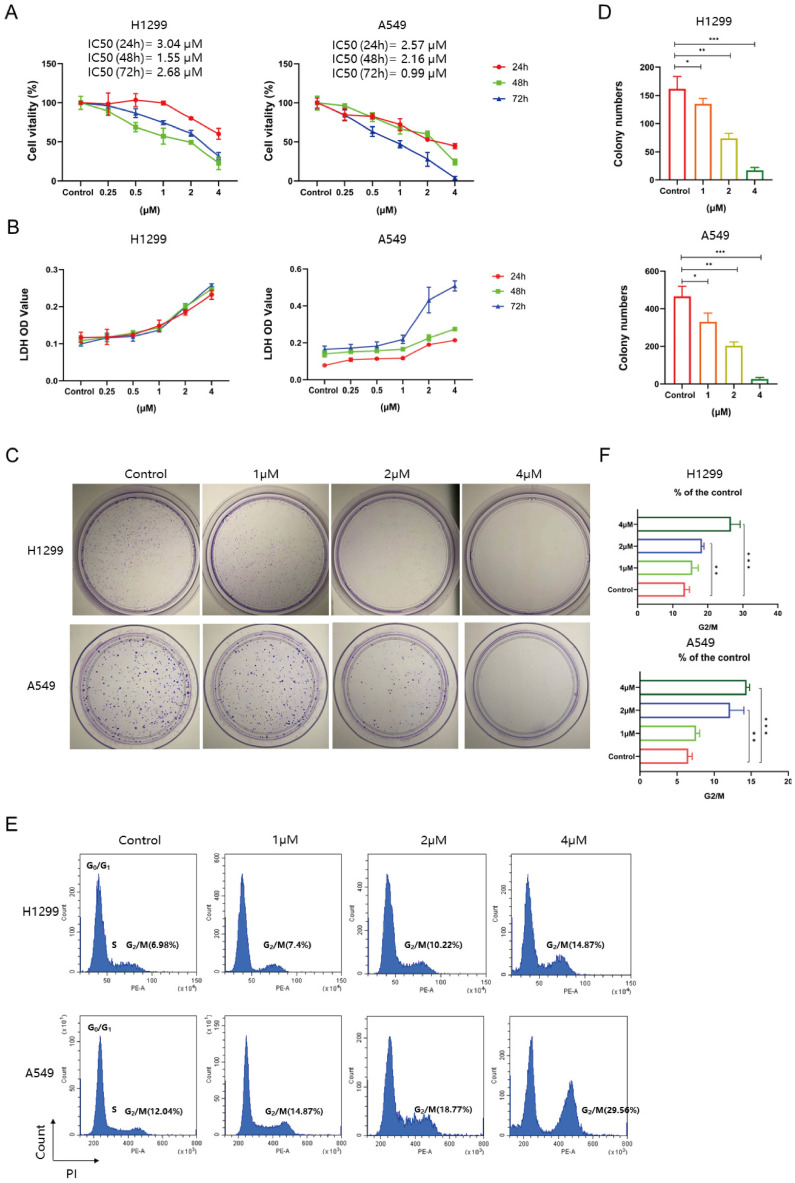
Tim-AIII inhibits proliferation, induces death, and leads to G2/M cycle arrest in NSCLC cells. **A** Viability of the H1299 and A549 cells was measured after treatment with different concentrations of Tim-AIII in different time periods by the CCK-8 assay. **B** The OD value of LDH in the supernatant of H1299 and A549 cells after treatment with different concentration of Tim-AIII in different time periods.** C** Representative results of monolayer culture in H1299 and A549 cells. **D** Quantitative analysis of colony numbers in H1299 and A549 cells. **E** Representative results of cell cycle in H1299 and A549 cells. **F** Quantitative analyzes of G2/M in H1299 and A549 cells. Quantitative data were presented as mean ± SD. ^*^p< 0.05, ^**^p < 0.01, ^***^p < 0.001.

To evaluate the cytotoxicity and inhibitory effects of Tim-AIII on NSCLC cells, H1299, A549, SPC-A1 and LLC cells were treated with different concentrations of Tim-AIII for diverse time periods. First, cell viability was analyzed using a CCK-8 assay. The results showed that Tim-AIII inhibited the growth of NSCLC cells in a dose- and time-dependent manner, and 90% of NSCLC cells died when treated with 4 μM Tim-AIII for 48 h (**Fig. 1A** and **Fig. S1A**). At concentrations up to 4 μM, Tim-AIII hardly affected the cell viability (90% of cells survived) of HBE cells (**Fig. S1B**). When further analyzing the level of LDH, the enzyme released from damaged or dead cells, we observed that Tim-AIII induced the release of LDH in a dose- and time-dependent manner for NSCLC cell lines (**Fig. 1B** and **Fig. S1C**). The colony formation assay was used to assess cell proliferation after Tim-AIII treatment. The results showed that Tim-AIII dose-dependently inhibited colony formation of both H1299 and A549 cells. Again, at the 4 μM Tim-AIII used, almost no colonies can be found (**Fig. 1C-D**). To further confirm the influence of Tim-AIII on cell proliferation, the cell cycle was examined by flow cytometry after Tim-AIII treatment. It showed that Tim-AIII promoted G2 / M phase arrest in a dose-dependent manner (**Fig. 1E-F**). Taken together, the results showed that Tim-AIII, by initiating G2/M-phase arrest, potently reduced cell proliferation and viability, even caused cell death in NSCLC cells.

### Tim-AIII suppresses the migration of NSCLC cells by inhibiting epithelial-mesenchymal transition

**Figure 2:**
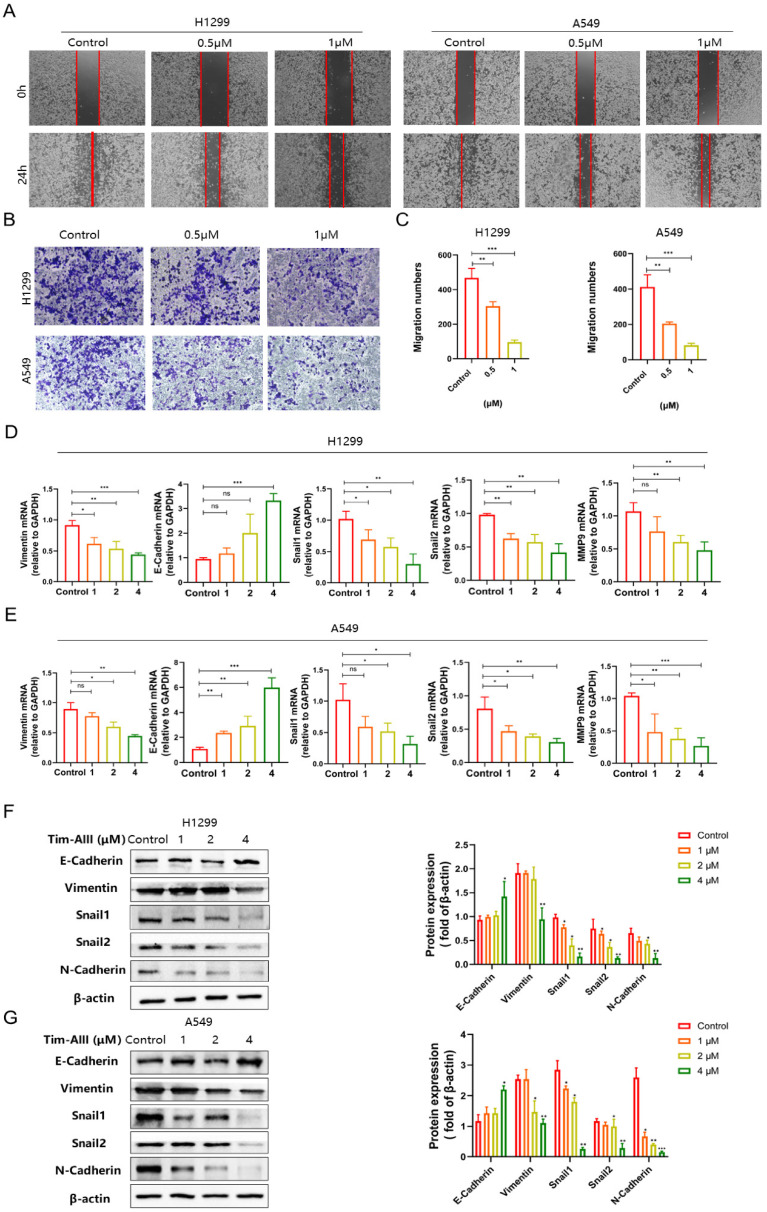
Tim-AIII suppresses the migration of NSCLC cells. **A** Representative result of wound healing assays in H1299 and A549 cells after treatment with Tim-AIII for 24 h. **B** Crystal violet stain of H1299 and A549 cells crystal violet stain (200×) after treatment with Tim-AIII for 24 h. **C** Quantification of the migration number of H1299 and A549 cells. **D, E** The expression of several migration-related genes, *VIM, E-Cadherin, SNAIL-2, MMP-9, SNAIL-1*, was examined by RT-PCR after treatment with Tim-AIII for 48 h in H1299 and A549 cells. **F** On the left side, the expression of various migration-related protein, E-cadherin, Vimentin, Snail-1, Snail-2, N-cadherin, was examined by WB after treatment with Tim-AIII for 48 h in H1299 and A549 cells. On the right is the statistics of appeal protein. Quantitative data were presented as mean ± SD. ^*^p< 0.05, ^**^p < 0.01, ^***^p < 0.001.

Cancer cell migration is fundamental for its metastasis. To investigate the effect of Tim-AIII on NSCLC cell migration, wound healing assay was performed. The results showed that treatment with quite low doses of Tim-AIII (0.5 μM) significantly inhibited H1299 and A549 cell migration compared to untreated controls (**Fig. 2A**). Next, a transwell assay was performed to further confirm the inhibitory effects on the migration of invasive cell migration. As shown in **Fig. 2B-C**, both 0.5 μM and 1 μM Tim-AIII significantly inhibited H1299 and A549 cell migration after Tim-AIII treatment for 24 h, while no significant effects were observed in the control groups.

Furthermore, the epithelial-mesenchymal transition (EMT) is known to play an important role in cancer progression, metastasis, and drug resistance. Here, we examined the expression level of several markers in NSCLC cells both in protein and mRNA levels after administration for 48 h. We found that Tim-AIII suppressed the expression level of mesenchymal marker genes of *VIM*, *SNAIL-2*, *SNAIL-1, MMP9* and increased the expression levels of epithelial marker genes of *E-Cadherin* (**Fig. 2D, E**). Consistent with the results of mRNA expression, we also found that the protein levels of vimentin, snail-1, snail-2, N-cadherin were also significantly negatively regulated, while the epithelial marker E-cadherin was positively regulated, indicating that Tim-AIII suppressed the EMT of NSCLC cells (**Fig. 2F**). These data suggest that Tim-AIII suppresses NSCLC cell migration mainly by inhibiting EMT.

### Ferroptosis was identified as a main determinant of Tim-AIII-induced cell death in NSCLC cells

**Figure 3:**
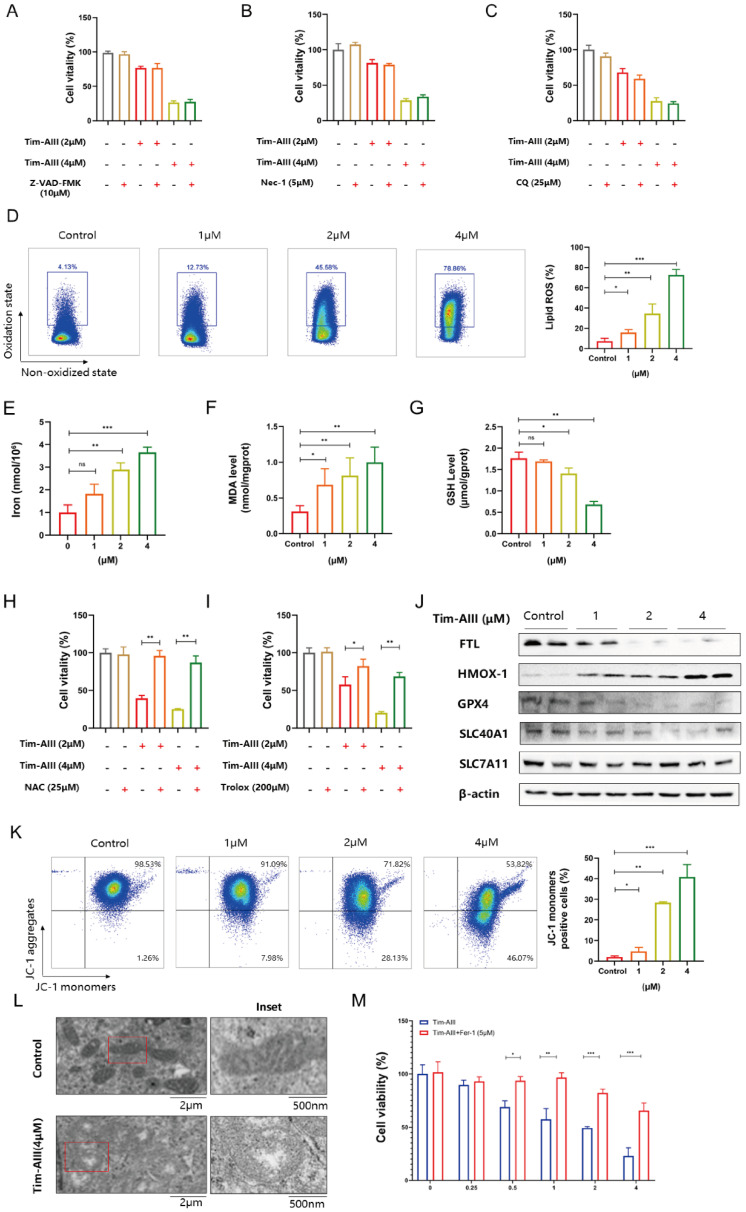
Tim-AIII-induced cell death is mainly caused by ferroptosis in H1299 cells. **A-C** H1299 cells were cotreated with Tim-AIII with or without Z-VAD-FMK, Nec-1 and CQ for 48 h, and cell vitality was assayed by the CCK-8 assay. **D** The lipid ROS level was analyzed using a flow cytometer after treatment with Tim-AIII for 48 h in H1299 cells. **E** The intracellular iron level after Tim-AIII treatment for 48 h in H1299 cells. **F** Intracellular MDA levels after treatment with Tim-AIII for 48 h in H1299 cells.** G** The intracellular GSH levels after Tim-AIII treatment for 48 h in H1299 cells. **H-I** H1299 cells were co-treated with Tim-AIII with or without the ROS inhibitor NAC and trolox for 48 h, and cell viability was assessed by CCK-8.** J** The expression of several key ferroptosis regulators, such as FLT, HMOX-1, GPX4, SLC40A1, SLC7A11, was examined by WB in H1299 cells. **K** Representative results of flow cytometry and quantification of mitochondrial membrane potential (JC-1 monomers) after treatment with Tim-AIII for 48 h in H1299 cells. **L** TME was used to observe mitochondrion in H1299 cells. **M** H1299 cells were cotreated with Tim-AIII with or without Fer-1 for 48 h, and cell vitality was assayed by the CCK-8 assay. Quantitative data were presented as mean ± SD. ^*^p< 0.05, ^**^p < 0.01, ^***^p < 0.001.

It is known that Tim-AIII exhibits the ability to suppress cell viability and induce cell death. We also asked which kind of cell death was triggered by Tim-AIII. To this end, several cell death inhibitors were applied in the coculture system. After co-treatment with Z-VAD-FMK (an apoptosis inhibitor) CQ (an autophagy inhibitor), or Nec-1 (a necroptosis inhibitor), Tim-AIII-induced cell death was not reversed both in H1299 cells (**Fig. 3A-C**) and A549 cells (**Fig. S2A-C**), suggesting that apoptosis, autophagy and necroptosis are not the main events triggered by Tim-AIII to induce cell death, therefore other kind of cell death may have occurred.

In recent years, more and more evidence has shown that ferroptosis plays an important role in tumor cell death 35. Therefore, we addressed whether ferroptosis is involved in Tim-AIII-induced cell death. Experimentally, we first analyze the levels of intracellular lipid ROS and iron, GSH, and MDA in NSCLC cells upon treatment with Tim-AIII, which are different parameters for quantifying ferroptosis. The data showed that ROS release, iron accumulation, MDA production, and GSH depletion were observed in H1299 (**Fig. 3D-G**) and A549 (**Fig. S2D-G**) after treatment with Tim-AIII for 48 h, indicating that ferroptosis was indeed triggered after Tim-AIII treatment. Furthermore, Tim-AIII-induced cell death were inhibited by cotreatment with the ROS inhibitor NAC or the antioxidant trolox as detected by the CCK-8 assay in H1299 cells (**Fig. 3H-I**) and A549 cells (**Fig. S2H-I**). Meanwhile, we detected the expression of a series of proteins closely related to the ferroptosis process in NSCLC cells upon Tim-AIII treatment. The data demonstrated that Tim-AIII treatment significantly increased the level of the pro-ferroptosis protein HMOX-1, but decreased the proteins that reverse ferroptosis, including FTL, GPX4, SLC40A1 and SLC7A11 in H1299 cells (**Fig. 3J**), A549 cells (**Fig. S2J**) and LLC cells (**Fig. S2K**). Furthermore, mitochondrial change is also be an important event in the process of ferroptosis 36-38. Therefore, we examined the mitochondria in Tim-AIII treated NSCLC cells by TEM and flow cytometry. MMP is a critical parameter for mitochondrial function and is usually visualized by JC-1 staining. In high MMP, JC-1 aggregates in the matrix of mitochondria and forms polymer (JC aggregates), which produces red fluorescence. When the mitochondrial membrane potential is low, JC-1 cannot aggregate in the mitochondrial matrix, and green fluorescence will appear. In fact, our data showed that MMP in H1299 cells (**Fig. 3K**) and A549 cells (**Fig. S2L**) decreased following treatment with Tim-AIII for 48 h, as showed by increased green fluorescence, indicating that Tim-AIII could reduce MMP and impair mitochondrial function. As a result, condensation of the mitochondrial matrix and formation of enlarged cristae were detected in both H1299 (**Fig. 3L**) and A549 cells (**Fig. S2M**) after treatment with Tim-AIII, but not in the untreated control group. Furthermore, Tim-AIII-induced cell death was blocked by cotreatment with different concentration of ferroptosis inhibitor ferrostatin-1 (Fer-1) in H1299 cells (**Fig. 3M**) and A549 cells (**Fig. S2N**). Taken together, these findings strongly suggested that Tim-AIII-induced cell death in NSCLC cells was achieved mainly by induction of ferroptosis.

### Tim -AIII-induced ferroptosis in NSCLC cells by targeting HSP90

**Figure 4:**
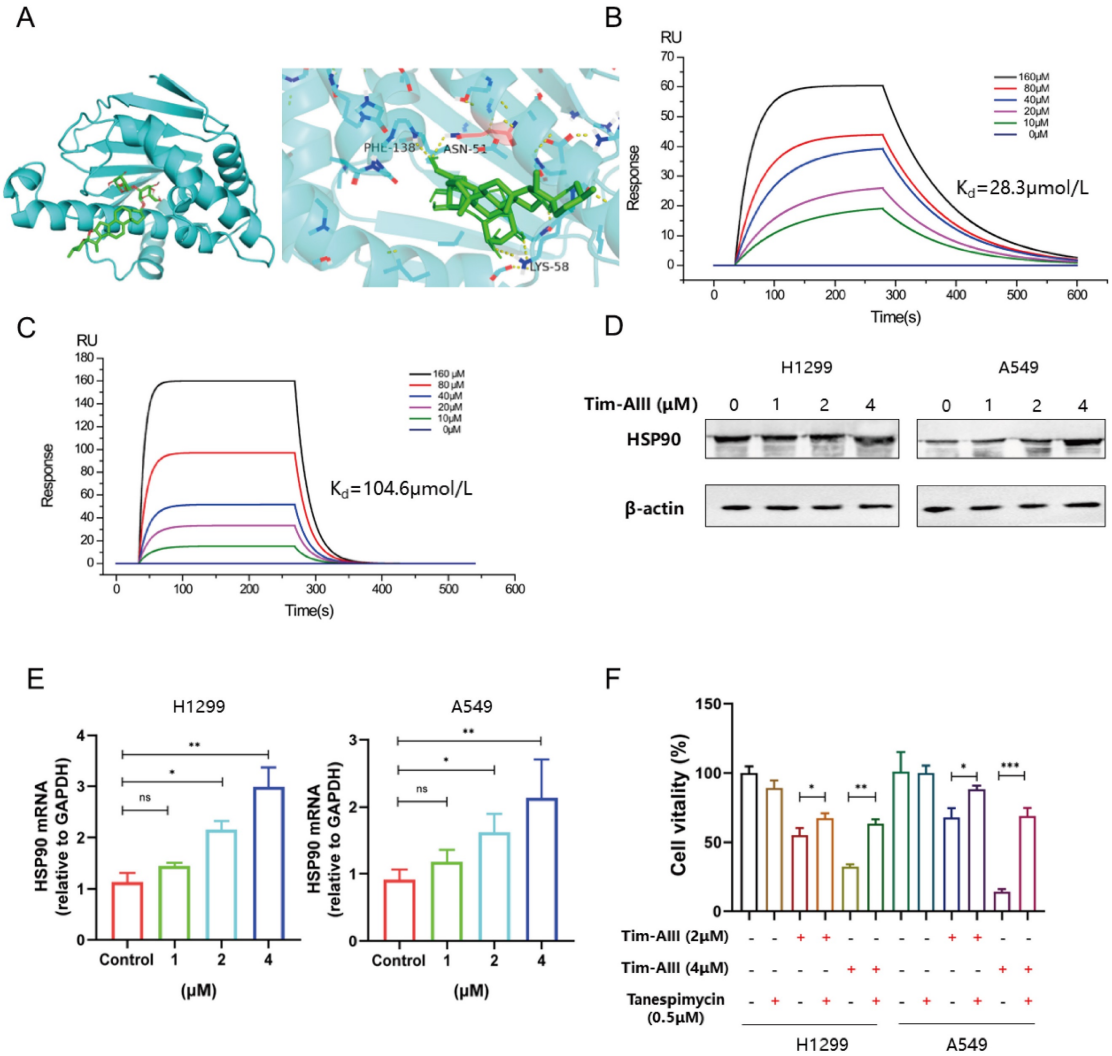
HSP90 is probably responsible for Tim-AIII-induced ferroptosis in NSCLC cells. **A** The most likely predicted targets of Tim-AIII in HSP90. B. SPR analysis of Tim-AIII binding to wild type HSP90 (K_d_ =28.2 μmmol/L). **C** SPR analysis of Tim-AIII binding to mutant HSP90 (K_d_=104.6 μmmol/L) **D** The protein expression of HSP90 was examined by WB after treatment with Tim-AIII for 48 h in H1299 and A549 cells. **E** The expression of *HSP90* were examined by RT-PCR after treatment with Tim-AIII for 48 h in H1299 and A549 cells. **F** H1299 and A549 cells were co-treated with Tim-AIII with or without the HSP90 classic inhibitor (tanespimycin) for 48 h, and cell vitality was assayed by the CCK-8 assay. Quantitative data were presented as mean ± SD. ^*^p< 0.05, ^**^p < 0.01, ^***^p < 0.001.

To explore the most likely target protein of Tim-AIII in the progression of ferroptosis, we used the online tool SwissTargetPrediction (STP) to make predictions. According to the STP results, the possible target of Tim-AIII was identified as HSP90 (**Fig. 4A**). HSP90 is a molecular chaperone that is conserved from bacteria to humans and facilitates the maturation of substrates (or clients) that are involved in different cellular pathways 39. Importantly, it has recently been reported that HSP90 chaperone-mediated autophagy promotes ferroptosis 40. To better verify whether HSP90 is a direct target of Tim-AIII, the interaction and viscosity coefficient between Tim-AIII and HSP90 were examined by a SPR assay. Our data showed that Tim-AIII readily bound to HSP90 with a K_d_ estimated at 28.3 μmmol/L (**Fig. 4B**). Next, the possible interaction motif within HSP90 for Tim-AIII binding was further investigated. First, a mutant HSP90 was prepared by site-directed mutagenesis to specifically mutate several highly active amino acids, which are ASN51, LYS58 and PHE138, respectively (**Fig. S3**). Then the binding affinity of Tim-AIII to the mutant HSP90 was further analyzed by SPR study. The data showed that the mutation of ASN51, LYS58 and PHE138 significantly decreased the binding affinity between HSP90 and Tim-AIII, as shown with a K_d_ estimated as 104.6 μmmol/L (**Fig. 4C**), indicating that HSP90 is a direct target of Tim-AIII and ASN51, LYS58, and PHE138 played key roles for HSP90-Tim-AIII interaction.

Knowing that HSP90 is a direct target for Tim-AIII, we next ask whether binding to HSP90 contributes to Tim-AIII-induced ferroptosis. To this end, both the protein and mRNA levels of HSP90 were first analyzed in NSCLC cells co-cultured with or without Tim-AIII for 48 h. As expected, HSP90 both increased both in the protein and mRNA levels when cells were treated with Tim-AIII (**Fig. 4D, E**). Next, Tim-AIII-induced ferroptosis was analyzed in the presence of the HSP90 inhibitor, the results showed that Tim-AIII-induced ferroptosis in NSCLC cells was blocked by cotreatment with tanespimycin, an inhibitor of HSP90 (**Fig. 4F**), which further proved that HSP90 plays a vital role in the process of Tim-AIII-induced ferroptosis. Taken together, these data revealed that HSP90 is a direct target of Tim-AIII, which functions as an important mediator in the Tim-AIII-mediated ferroptosis process.

### Ferroptosis induced by the Tim-AIII-HSP90 complex in NSCLC cells was achieved by targeting, ubiquitinating and degrading GPX4

**Figure 5:**
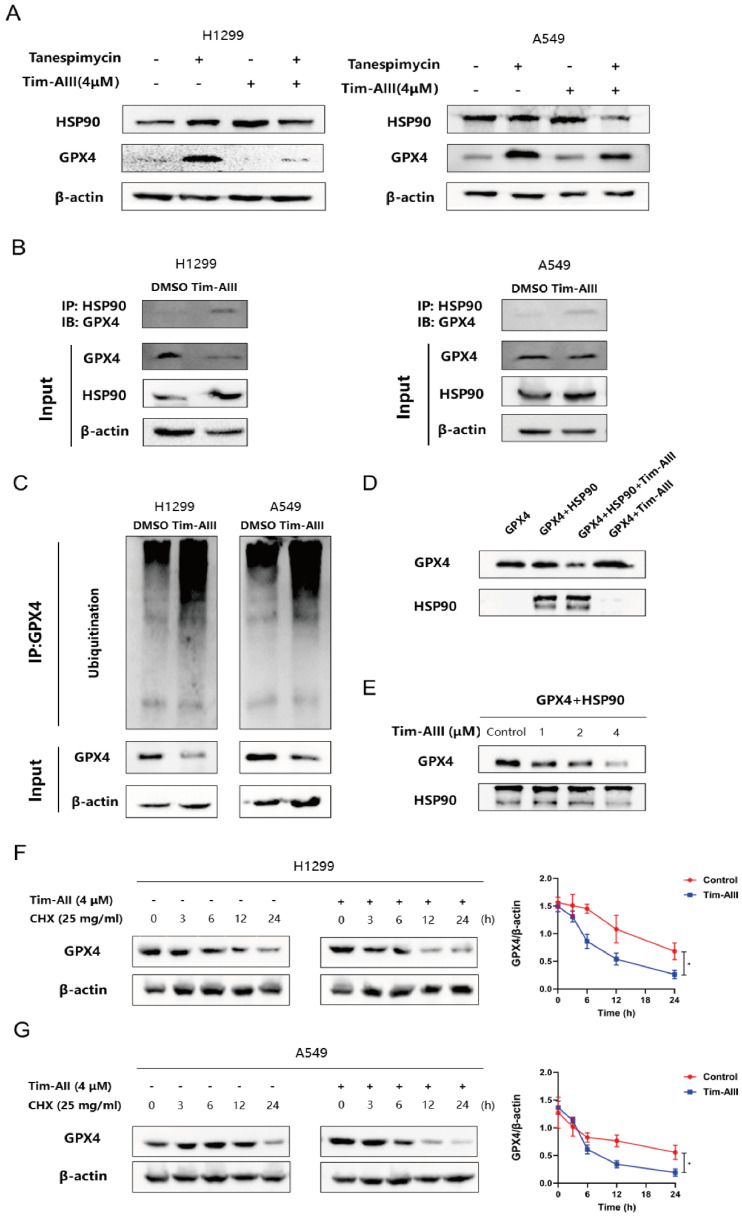
Tim-AIII-HSP90 complex-induced ferroptosis in NSCLC cells was achieved by targeting and degrading GPX4. **A** The protein expression of HSP90 and GPX4 was examined by WB after treatment with Tim-AIII with or without tanespimycin for 48 h in H1299 and A549 cells. **B** Immunoprecipitation was performed to detect the interaction between GPX4 and HSP90, and WB was performed to detect HSP90 levels in the inputs and immunoprecipitates in treatment with Tim-AIII (4 μM) for 48 h in H1299 and A549 cells.** C** Tim-AIII was added into H1299 and A549 cells and incubated for 48 h. Then, 10 μM MG-132 was added to inhibit the protein degradation for 4 h. The proteins were collected and incubated GPX4 antibody level of shaking 4 ℃ overnight, then agarose G was added and co-incubated at 4 °C for 3h. The ubiquitination level of GPX4 was analyzed by WB assay with anti-Ubiquitination antibody. **D** The expression of GPX4 was examined by WB after incubating with HSP90 in the presence or absence of Tim-AIII (4 μM). **E** The protein expression of the GPX4 were examined by WB after incubation with HSP90 on the usage of different amounts of Tim-AIII. **F** WB analysis of the GPX4 expression treated with DMSO or Tim-AIII (4 μM) for the indicated time points in the presence of CHX (25 mg/mL) in H1299 cells on the left side. Quantification of GPX4 intensity, the abundance was normalized to β-actin on the right side. **G** WB analysis of the GPX4 expression treated with DMSO or Tim-AIII (4 μM) for the indicated time points in the presence of CHX (25 mg/mL) in A549 cells on the left side. Quantification of GPX4 intensity, the abundance was normalized to β-actin on the right side. Quantitative data were presented as mean ± SD. ^*^p< 0.05, ^**^p < 0.01, ^***^p < 0.001.

Due to the inhibitory role of GPX4 in ferroptosis, here we further addressed whether Tim-AIII could show any effects on GPX4. As shown above in our co-culture system, the level of GPX4 in H1299 cells (**Fig. 3L**), A549 cells (**Fig. S2J**), and LLC cells (**Fig. S2K**) was much lower when cells were treated with Tim-AIII. However, Tim-AIII-mediated reduction of GPX4 was reversed when cotreated with HSP90 inhibitor tanespimycin (**Fig. 5A**), indicating that HSP90 was involved in Tim-AIII-triggered reduction of GPX4 in NSCLC cells, and there might be a triple interaction among Tim-AIII, HSP90, and GPX4. To prove this hypothesis, immunoprecipitation was performed to investigate the interaction between HSP90 and GPX4 in the presence or absence of Tim-AIII. The results showed that Tim-AIII administration increased the mutual combination of HSP90 and GPX4, which in turn probably further triggered GPX4 degradation in NSCLC cells (**Fig. 5B**). Ubiquitination is an enzymatic process involving the binding of ubiquitin proteins to substrate proteins. The most common result of ubiquitination is protein degradation by proteasome. Therefore, we analyzed the ubiquitination of GPX4 after Tim-AIII treatment. The results show that Tim-AIII could increase the ubiquitination of GPX4 (**Fig. 5C**). To further prove and investigate the degradation mediated by the Tim-AIII-HSP90 complex of GPX4, HSP90 were incubated with GPX4 in the presence or absence of different amounts of Tim-AIII. Therefore, GPX4 degradation was therefore quantified by WB using an anti-GPX4 antibody. The data showed that the Tim-AIII (4 μM)-HSP90 complex could significantly degrade GPX4 (**Fig. 5D**). And, GPX4 degradation was dose-dependent on the usage of Tim-AIII (**Fig. 5E**). In order to further verify that the Tim-AIII-HSP90 complex promoted the ubiquitination and degradation of GPX4 rather than inhibited the translation process of GPX4. NSCLC cells were exposed to cycloheximide (CHX), a protein translation inhibitor with or without Tim-AIII. CHX pulse tracking analysis showed that the transformation rate of GPX4 in cells treated with Tim-AIII was faster than that in untreated cells (**Fig. 5F, G**), indicating that Tim-AIIII regulated GPX4 expression at the post translation level. Taken together, these data showed that HSP90, by forming a complex with Tim-AIII, further targeted and triggered GPX4 ubiquitination and degradation, which in turn induced ferroptosis in NSCLC cells.

### Ferroptosis induced by the Tim-AIII was dependent on HSP90

**Figure 6:**
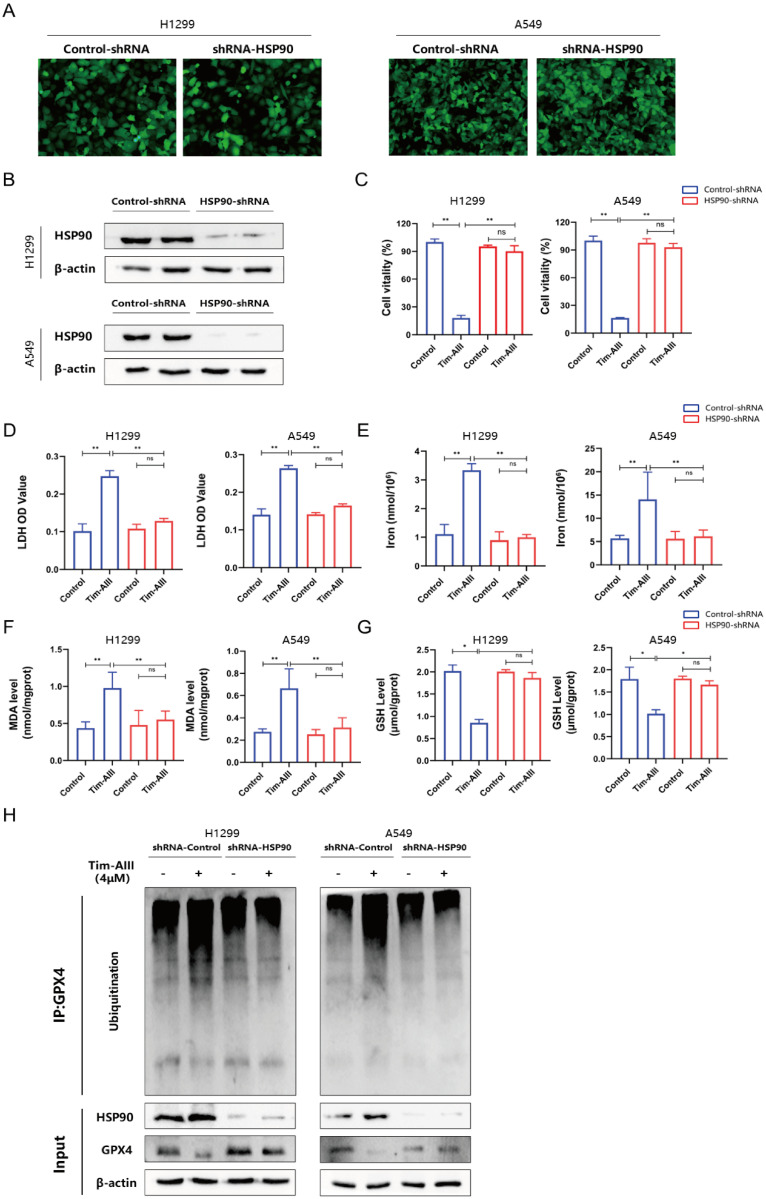
** Ferroptosis induced by the Tim-AIII was dependent on HSP90. A** The transfection efficiencies of HSP90-shRNA in H1299 and A549 cells were evaluated by observing the fluorescence of GFP (200X). **B** The expression of HSP90 protein in H1299 and A549 cells after transfection was detected by WB. **C** H1299 and A549 cells were transfected with control-shRNA and HSP90-shRNA, followed by Tim-AIII (4 μM) treatment for 48 h, and cell vitality was assayed by the CCK-8 assay. D H1299 and A549 cells were transfected with control-shRNA and HSP90-shRNA, followed by Tim-AIII (4 μM) treatment for 48 h, and the OD value of LDH was assayed. **E-G** H1299 and A549 cells were transfected with control-shRNA and HSP90-shRNA, followed by Tim-AIII (4 μM) treatment for 48 h, and the level of intracellular iron, MDA and GSH were assayed.** H** H1299 and A549 cells were transfected with control-shRNA and HSP90-shRNA, followed by Tim-AIII (4 μM) treatment for 48 h, the GPX4, HSP90 and the ubiquitination level of GPX4 were analyzed by WB assay with anti-Ubiquitination antibody. Quantitative data were presented as mean ± SD. ^*^p< 0.05, ^**^p < 0.01, ^***^p < 0.001.

Based on the appeal results, Tim-AIII targeted HSP90 and induced GPX4 ubiquitination and degradation. Next, we generated the HSP90 knockdown models in NSCLC cell lines H1299 and A549 through transfecting with HSP90-shRNA. In **Fig. 6A-B**, representative images revealed that HSP90 levels in H1299 and A549 cells transfected with HSP90-shRNA were evidently lower than those transfected with control-shRNA. The inhibition of proliferation of H1299 and A549 cells by Tim-AIII was markedly abrogated when HSP90 was knocked down by HSP90-shRNA transfection (**Fig. 6C**). Tim-AIII induced the release of LDH in H1299 and A549 cells was markedly abrogated when HSP90 was knocked down by HSP90-shRNA transfection (**Fig. 6D**). Tim-AIII led to iron accumulation, MDA production, and GSH depletion in H1299 and A549 cells were all markedly abrogated when HSP90 was knocked down by HSP90-shRNA transfection (**Fig. 6E-G**). In addition, Tim-AIII-mediated GPX4 ubiquitination and degradation were abrogated when HSP90 was knocked down by HSP90-shRNA transfection (**Fig. 6H**).

### Tim-AIII exerts antitumor efficacy by inducing ferroptosis *in vivo*

**Figure 7:**
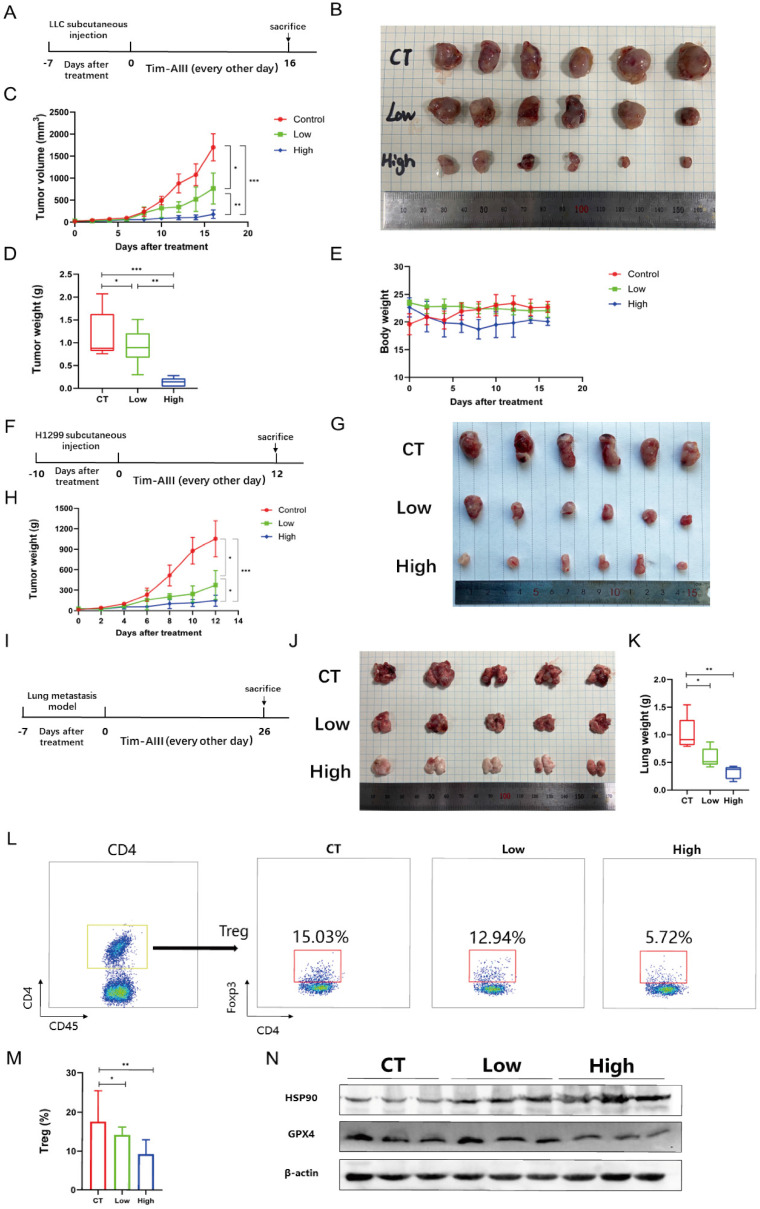
Tim-AIII suppresses tumor progression *in vivo*. **A** C57BL/6J mice that carried tumor derived from LLC cells subcutaneous xenografts tumor were administered Tim-AIII (low dose: 12.5 mg/kg or high dose: 50 mg/kg) or PBS every other day. **B** Representative image of the subcutaneous tumor of xenografts in different groups of C57BL/6J mice. **C** The tumor curves of C57BL/6J mice in different groups. D The weights of the tumor derived from LLC cells subcutaneous xenografts tumor. **E** Body weights of the C57BL/6J mice during the experimental period. **F** BALB/c-nu/nu nude mice that carried tumor derived from H1299 cells subcutaneous xenografts tumor were administered Tim-AIII (low dose: 12.5 mg/kg or high dose: 50 mg/kg) or PBS every other day. **G** Representative image of the subcutaneous tumor of xenografts in different groups of BALB/c-nu/nu nude mice. H The tumor curves of BALB/c-nu/nu nude mice in different groups. **I** C57BL/6J mice with LLC cells-derived lung metastasis tumor through the tail vein were intraperitoneal injection administered intraperitoneally Tim-AIII (Low dose: 12.5 mg/kg or high dose: 50 mg/kg) or PBS every other day. **J** Representative image of the lung of C57BL/6J mice in different groups. **K** The weights of the lung of C57BL/6J mice in different groups. **L** Representative flow cytometry results of Treg cells in the tumor microenvironment of LLC cells derived subcutaneous xenografts tumor microenvironment. **M** Quantitative analyses of Treg cells in LLC cells-derived subcutaneous xenografts. **N** The protein expression of HSP90 and GPX4 was examined by WB in LLC cells-derived subcutaneous xenografts tumor. Quantitative data were presented as mean ± SD. ^*^p< 0.05, ^**^p < 0.01, ^***^p < 0.001.

To estimate the therapeutic potential of Tim-AIII in *vivo*, we used two different tumor models, subcutaneous xenograft tumor model and lung metastasis tumor model. C57BL/6J mice were first subcutaneously injected with LLC cells, then randomly divided into three experimental groups after 7 days after injection: control group (CT), the low-dose Tim-AIII group (low, 12.5 mg / kg) and the high-dose Tim-AIII group (high, 50 mg / kg) (**Fig. 7A**). Tim-AIII treatment markedly suppressed tumor growth in a dose-dependent manner verified by tumor volume and tumor weight compared with controls (**Fig. 7B-D**), while there was no significant difference in mouse body weight (**Fig. 7E**). In addition, BALB/c-nu/nu nude mice were subcutaneously injected with H1299 cells, then randomly divided into three experimental groups after 10 days after injection: control group (CT), the low-dose Tim-AIII group (low, 12.5 mg / kg) and the high-dose Tim-AIII group (high, 50 mg / kg) (**Fig. 7F**). Tim-AIII treatment markedly suppressed tumor growth in a dose-dependent manner compared with controls (**Fig. 7G, H**). Then, a lung metastasis tumor model was applied. LLC cells were injected intravenously into C57BL/6J mice, and was randomly divided into three experimental groups and administered Tim-AIII or PBS for 26 days: the control group (CT), low-dose Tim-AIII treated group (low, 12.5 mg / kg), and the high-dose Tim-AIII (high, 50 mg / kg) (**Fig. 7I**). Again, Tim-AIII treatment significantly suppressed lung tumor when compared to controls (**Fig. 7J, K**). Meanwhile, to assess Tim-AIII side effects following the administration in vivo, various organs and blood were harvested from the above treated mice. Organs were sectioned and stained with H&E. No histological differences were detected in the liver, heart, spleen, lung, or kidney between the high-dose Tim-AIII treated group and the control group, indicating that there was no significant toxicity (**Fig. S4A**). Next, plasma was prepared and the ALT, AST, Cre and BUN plasma levels were analyzed. Similarly, no significant differences were detected for ALT, AST, Cre and BUN between the control group and the high-dose Tim-AIII treated group (**Fig. S4B-C**). Furthermore, we examine immune cells in subcutaneous xenograft tumors. Flow cytometry results showed that the percentage of Treg cells decreased two-three times in tumor microenvironment after receiving high-dose Tim-AIII treatment (**Fig. 7L, M**), indicating that the Treg-mediated immunosuppressed condition in the tumor microenvironment was changed after Tim-AIII treatment. Next, we detected the protein levels of HSP90 and GPX4 in subcutaneous xenograft tumors. Indeed, HSP90 expression significantly increased and GPX4 expression decreased after treatment with different doses of Tim-AIII (**Fig. 7N**), which is consistent with the *in vivo* data.

### Models of Tim-AIII meditated anticancer effects via induction of ferroptosis

**Figure 8:**
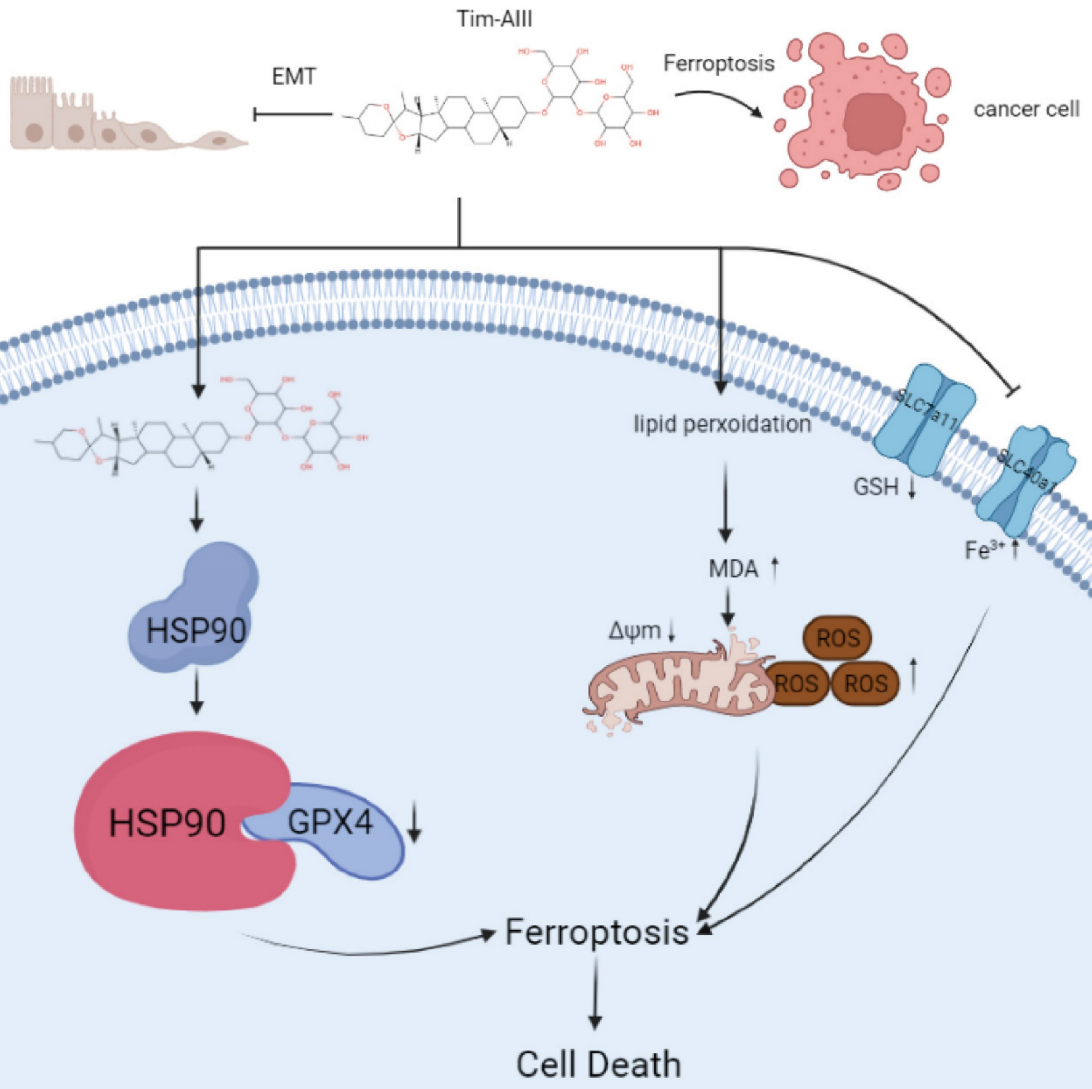
Scheme showing the central role of Tim-AIII in ferroptosis induction and inhibition of cell migration.

In this study, we performed a series of experiments to explore the effect and underlying mechanism of Tim-AIII in NSCLC. Our data reveal that Tim-AIII enters cells and binds to HSP90 to form a complex. The Tim-AIII-HSP90 complex then further interacts and degrades GPX4, which ultimately leads to the ferroptosis process (**Fig. 8**). Meanwhile, Tim-AIII also causes ROS and iron accumulation, lipid peroxidation, GSH depletion, MMP reduction, of reduction and solute carrier family proteins (SLC40A1 and SLC7A11) (**Fig. 8**) which typically occurs during ferroptosis. Therefore, Tim-AIII meditated on anticancer effects via induction of ferroptosis in NSCLC.

## Discussion

Previous studies have shown that Tim-AIII, a steroidal saponin product derived from* Anemarrhena Asphodeloides Bunge*, has anticancer activity 28, 32, 33. However, the role of Tim-AIII in lung cancer remains unclear. Here, we analyzed the effects of Tim-AIII on NSCLC and further investigated the underlying mechanism *in vitro* and *in vivo*, and found that Tim-AIII exerts its anticancer activity mainly by inducing ferroptosis, which in turn caused the arrest of G2 / M phase, and inhibition of the proliferation and migration of NSCLC cells.

The goal of traditional cancer therapy is to induce apoptosis of cancer cells, but many cancer cells are chemoresistant or defective in induction of apoptosis. Therefore, the development of new drugs capable of inducing different forms of non-apoptotic cell death is urgently needed to provide a promising therapeutic strategy for cancer patients. Ferroptosis is an iron-dependent cell death that is morphologically, biochemically, and genetically distinct from apoptosis 19. Currently, ferroptosis-inducing drugs attract more attention for cancer treatment and will hopefully provide a potential strategy for cancer therapy 41, 42. Our data showed that cotreatment with Fer-1, an ferroptosis inhibitor, reduced Tim-AIII-induced cell death, indicating that ferroptosis contributes to Tim-AIII-induced cell death in NSCLC. However, cotreatment with the apoptosis inhibitor, autophagy inhibitor, or necroptosis inhibitor did not show any protective effects on cell viability, suggesting that apoptosis, autophagy, and necroptosis are not the main events in Tim-AIII-induced cell death. Additionally, treatment with Tim-AIII significantly induced lipid ROS and iron accumulation, GSH depletion, lipid peroxidation, and decreased mitochondrial membrane potential, further indicating that ferroptosis was induced after treatment with Tim-AIII.

GPX4 uses GSH to detoxify lipid peroxidation and plays an essential role in inhibiting ferroptosis. It is known that GPX4 specifically catalyzes the oxidative activity of lipid peroxides in a glutathione-dependent manner, thus protecting cells from the threat of ferroptosis*.* As an enzyme, it is crucial in the conversion of toxic lipid hydroperoxides into non-toxic lipid alcohols, GPX4 has been regarded as a recognized ferroptosis gatekeeper 12, 18. Interestingly, Tim-AIII treatment caused a reduction of GPX4 levels, indicating that GPX4 is likely involved in Tim-AIII-induced ferroptosis.

In this work, for the first time, HSP90 was identified as the direct target for Tim-AIII. Tim-AIII strongly binds to HSP90 with a K_d_ of 28.3 μmmol/L mainly through ASN51, LYS58, and PHE138. HSP90 plays a significant role in essential cellular processes and regulatory pathways such as apoptosis, cell cycle control, cell signaling, cell viability, protein folding, and degradation, as they interact with client proteins and co-chaperones 43. HSP90 is a vital chaperone protein conserved in all organisms. As a chaperone protein, it correctly folds client proteins 44. A recent study showed that chaperone-mediated autophagy is involved in the execution of ferroptosis, and HSP90 acts as a common regulatory mediator shared by necroptosis and ferroptosis 40. Our further mechanical analysis showed that there are triple interactions of Tim-AIII, GPX4, and HSP90 in the process of ferroptosis. In other words, Tim-AIII treatment increased HSP90 expression using an unknown strategy. HSP90 up-regulated functions as a direct target of Tim-AIII, interacts and forms a complex with Tim-AIII. The Tim-AIII-HSP90 complex, in turn, interacts with GPX4, thus inhibiting the function of GPX4, likely by further ubiquitination and degradation of GXP4, and further inducing ferroptosis.

As a new form of regulated cell death, ferroptosis is initiated by lipid peroxidation caused by ROS generation and iron overload. Therefore, the molecules that directly or indirectly affect these mediators will inevitably regulate the ferroptosis process. GSH is a critical player in the antioxidant system to prevent lipid peroxidation, and SLC7A11-mediated cystine uptake will increase GSH synthesis. Furthermore, the iron transporter SLC40A1 controls intestinal iron absorption and cellular iron release. Hence, alteration of GSH and solute carrier family proteins (SLC7A11 and SLC40A1) is always used to evaluate ferroptosis. In this study, we observed the ability of Tim-AIII to promote ROS and iron accumulation, lipid peroxidation, GSH depletion, MMP reduction, of reduction and solute carrier family proteins (SLC40A1 and SLC7A11), leading to ferroptosis.

The application of the *in vivo* model showed that Tim-AIII inhibited the progression of subcutaneous xenograft tumors and lung metastases tumors without apparent toxicity. Furthermore, increased HSP90 and the decreased GPX4 levels were detected in subcutaneous xenograft tumors after Tim-AIII administration, which further confirmed HSP90 and GPX4 were the main players involved in the anticancer effects triggered by Tim-AIII. Furthermore, we also found that the administration of Tim-AIII reduced the percentage of Treg cells in the subcutaneous xenograft tumor microenvironment, suggesting the multi-function of Tim-AIII in preventing tumor progression. It is known, that Treg cells are able to promote tumor progression by controlling immune responses initiated by T cells, B cells, natural killer cells (NK), dendritic cells (DCs), and macrophages in the tumor microenvironment 45. Therefore, Tim-AIII probably changed the immune microenvironment from anti-immune to pro-immune response condition, facilitating immune clearance of tumor cells.

It was reported that Tim-AIII at high concentrations from 10 μM to 30 μM induced both autophagy and apoptosis in A549 cells for 12 h. However, Tim-AIII at low concentration (1 μM) only induced autophagy in A549 cells for 12 h 46. It was also reported that Tim-AIII at a concentration of 9 μM could induce apoptosis in A549 cells for 24h 47. In our study, 90% of NSCLC cells died of ferroptosis when treated with 4 μM Tim-AIII for 48 h. Generally speaking, small molecule compounds may have multiple targets. For different cell death modes induced by Tim-AIII, we hypothesized that its key targets would change with the time and concentration.

In summary, our data provided compelling evidence demonstrating that Tim-AIII promotes NSCLC ferroptosis by targeting and facilitating HSP90-mediated GPX4 ubiquitination and degradation, which in turn causes cell cycle arrest, inhibition of cell proliferation, and migration. In addition, high therapeutic efficacy, low toxicity, and high selectivity shown in *in vivo* mouse modes prove that Tim-AIII serves as a potential compound for NSCLC therapy. However, the therapeutic effects of Tim-AIII in clinical trials need to be further investigated in the near future.

## Supplementary Material

Supplementary figures.

## Figures and Tables

**Table 1:** Primer sequences used for quantitative real-time PCR

β-actin	(forward) 5′-CACCATTGGCAATGAGCGGTTC-3′
	(reverse) 5′-AGGTCTTTGCGGATGTCCACGT-3′
GAPDH	(forward) 5′-ATGGGTGTGAACCACGAGA-3′
	(reverse) 5′-CAGGGATGATGTTCTGGGCA-3′
VIM	(forward) 5′-AGGCAAAGCAGGAGTCCACTGA-3′
	(reverse) 5′-ATCTGGCGTTCCAGGGACTCAT-3′
E-Cadherin	(forward) 5′-GCCTCCTGAAAAGAGAGTGGAAG-3′
	(reverse) 5′-TGGCAGTGTCTCTCCAAATCCG-3′
SNAIL-2	(forward) 5′-ATCTGCGGCAAGGCGTTTTCCA-3′
	(reverse) 5′-GAGCCCTCAGATTTGACCTGTC-3′
MMP9	(forward) 5′-GCCACTACTGTGCCTTTGAGTC-3′
	(reverse) 5′-CCCTCAGAGAATCGCCAGTACT-3′
SNAIL-1	(forward) 5′-TGCCCTCAAGATGCACATCCGA-3′
	(reverse) 5′-GGGACAGGAGAAGGGCTTCTC-3′
HSP90	(forward) 5′-TCTGCCTCTGGTGATGAGATGG-3′
	(reverse) 5′-CGTTCCACAAAGGCTGAGTTAGC-3′

## Abbreviations

Tim-AIII: Timosaponin AIII
HSP90: heat shock protein 90
GPX4: glutathione peroxidase 4
NSCLC: non-small cell lung cancer
ROS: reactive oxygen species
GSH: glutathione
MDA: malondialdehyde
FTL: Ferritin light chain
HMOX-1: Heme oxygenase 1
SLC40A1: Solute carrier family 40, member 1
SLC7A11: Solute carrier family 7, member 11
