# Supplementary material for: Timosaponin AIII promotes non-small-cell lung cancer ferroptosis through targeting and facilitating HSP90 mediated GPX4 ubiquitination and degradation
Source: Int J Biol Sci. 2023 Feb 27;19(5):1471–89. doi: 10.7150/ijbs.77979 (PMC10086754; doi:10.7150/ijbs.77979)
Supplement: Supplementary file 1 — Supplementary figures. [file ijbsv19p1471s1.pdf]

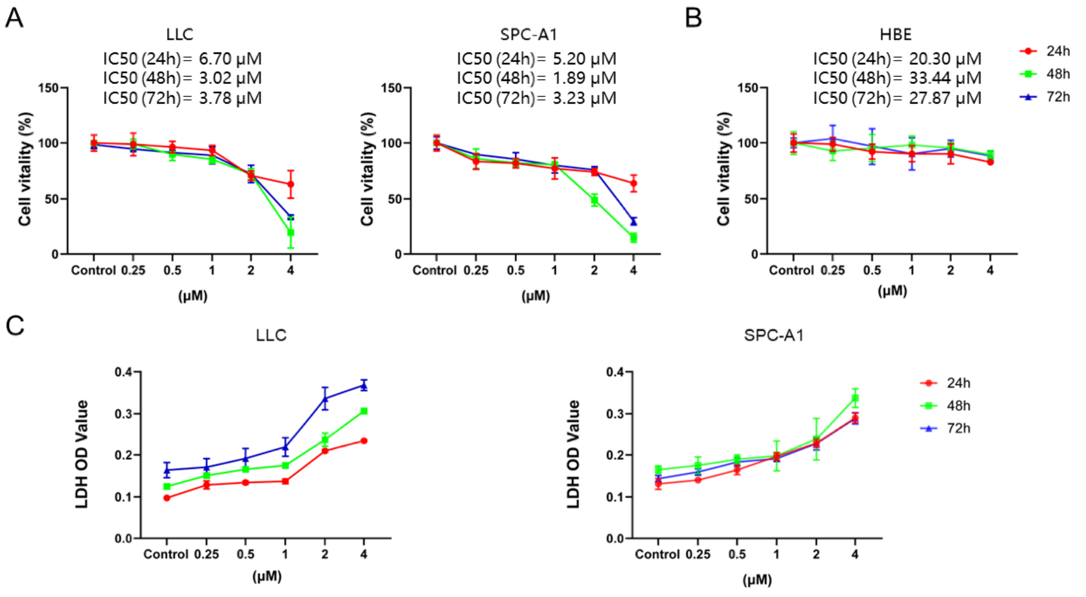

Fig.S1 Tim-AIII inhibits cell proliferation and induces cell death in LLC and SPC-A1 cells. **A** The LLC and SPC-A1 cells viability was measured after treatment with different concentration of Tim-AIII in different time periods. **B** The OD value of LDH in the supernatant of LLC and SPC-A1 cells after treatment with different concentration of Tim-AIII in different time periods. Quantitative data were presented as mean ± SD. $^{*}p< 0.05$, $^{**}p < 0.01$, $^{***}p < 0.001$.

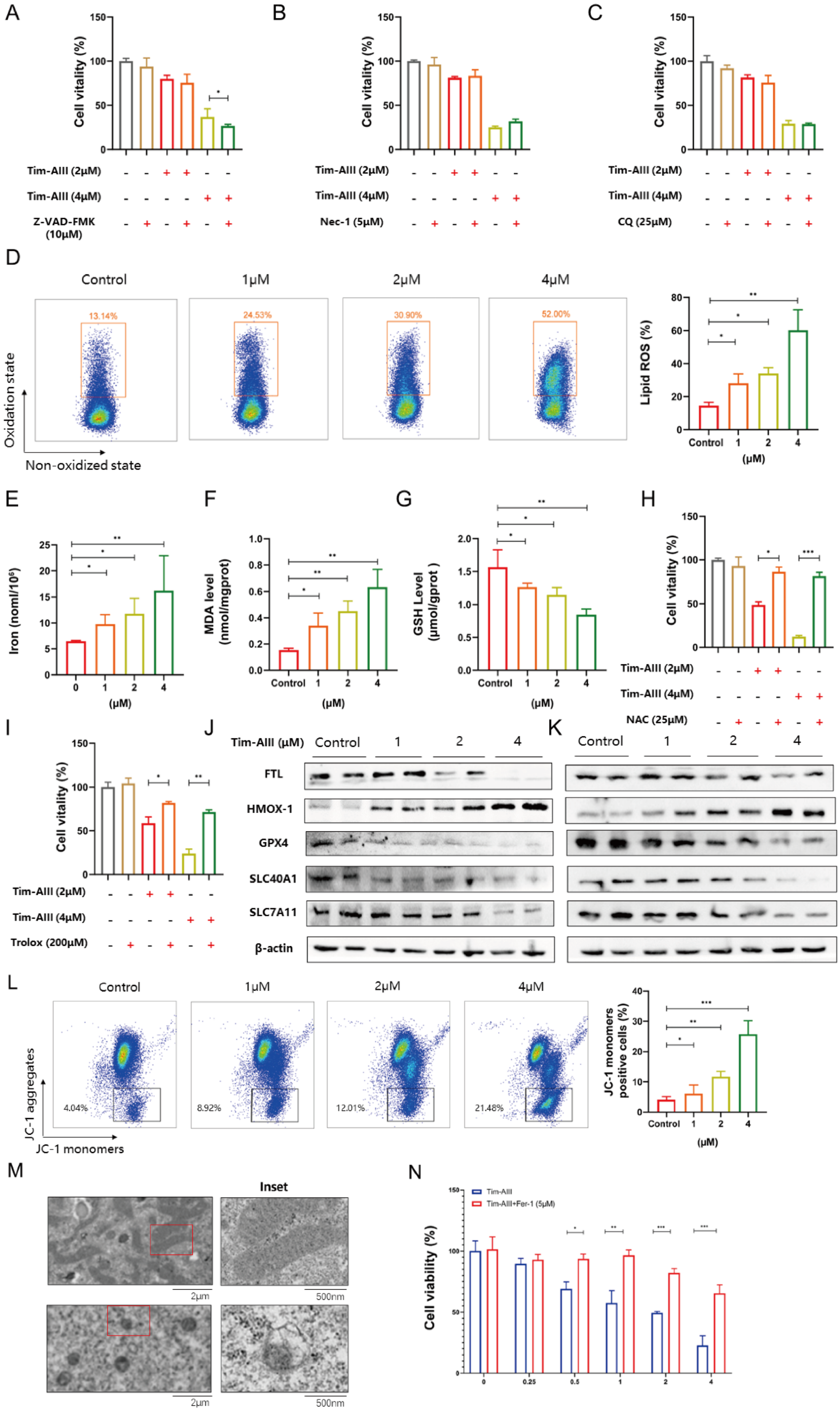
A
B
C
Cell vitality (%)
Tim-AIII (2μM)
Tim-AIII (4μM)
Z-VAD-FMK (10μM)
Nec-1 (5μM)
CQ (25μM)
D
Control
1μM
2μM
4μM
13.14%
24.53%
30.90%
52.00%
Oxidation state
Non-oxidized state
Lipid ROS (%)
(μM)
E
Iron (noml/10⁶)
F
MDA level (nmol/mgprot)
G
GSH Level (μmol/gprot)
H
NAC (25μM)
I
Trolox (200μM)
J
Tim-AIII (μM)
FTL
HMOX-1
GPX4
SLC40A1
SLC7A11
β-actin
K
L
4.04%
8.92%
12.01%
21.48%
JC-1 aggregates
JC-1 monomers
JC-1 monomers positive cells (%)
M
Inset
2μm
500nm
N
Tim-AIII
Tim-AIII+Fer-1 (5μM)
Cell viability (%)

Fig. S2 Tim-AIII-induced cell death is mainly caused by ferroptosis in A549 cells. **A-C** A549 cells were cotreated with Tim-AIII with or without Z-VAD-FMK, Nec-1 and CQ for 48h, and the cell vitality was assayed by CCK-8 assay. **D** The lipid ROS level was analyzed by a flow cytometer after treatment with Tim-AIII for 48h in A549 cells. **E** The intracellular iron level after treatment with Tim-AIII for 48h in A549 cells. **F** The intracellular MDA level after treatment with Tim-AIII for 48h in A549 cells. **G** The intracellular GSH level after treatment with Tim-AIII for 48h in A549 cells. **H-I** A549 cells were cotreated with Tim-AIII with or without the ROS inhibitor NAC and Trolox for 48h, and cell viability was assayed by CCK-8. **J** The expression of several key ferroptosis regulators, such as FLT, HMOX-1, GPX4, SLC40A1, SLC7a11, were examined by western blot in A549 cells. **K** The expression of several key ferroptosis regulators, such as FLT, HMOX-1, GPX4, SLC40A1, SLC7a11, were examined by western blot in LLC cells. **L** Representative flow cytometry results and quantification of mitochondrial membrane potential (JC-1 green) after treatment with Tim-AIII for 48h in A549 cells. **M** TME was used to observe mitochondrion in A549 cells. **N** A549 cells were cotreated with Tim-AIII with or without Fer-1 for 48h, and the cell vitality was assayed by CCK-8 assay. Quantitative data were presented as mean ± SD. $^{*}\boldsymbol{p}< 0.05$, $^{**}\boldsymbol{p} < 0.01$, $^{***}\boldsymbol{p} < 0.001$.

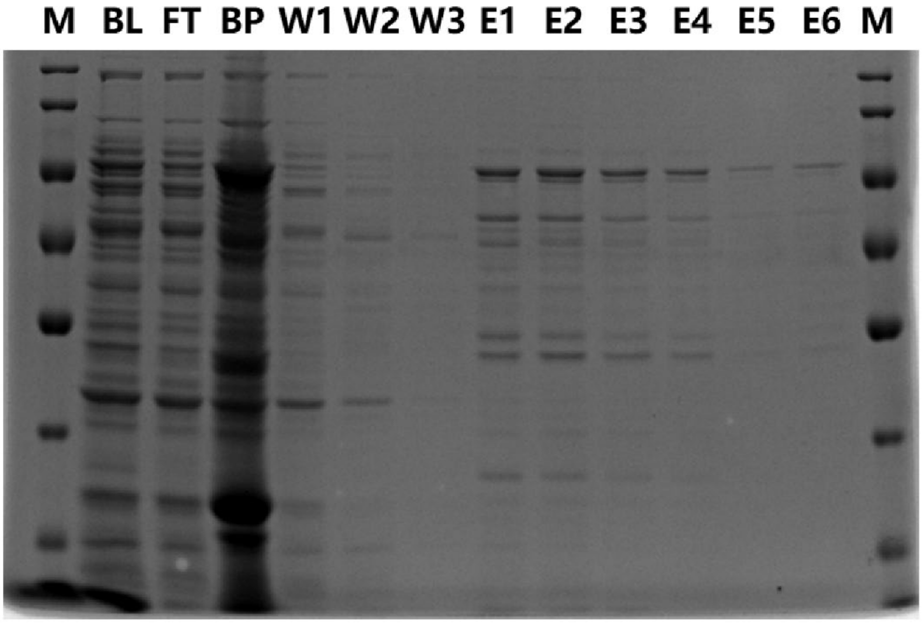

Fig. S3 Coomassie brilliant blue staining effect of purified recombinant HSP90 mutant protein with His tag. M: Marker; BL: bacterial lysate; FT: sample filling fluid; BP: bacterial precipitation;W1-W3: cracking liquid washing 1-3 (Wash 1-3); E1-E6: The eluate contains purified protein.

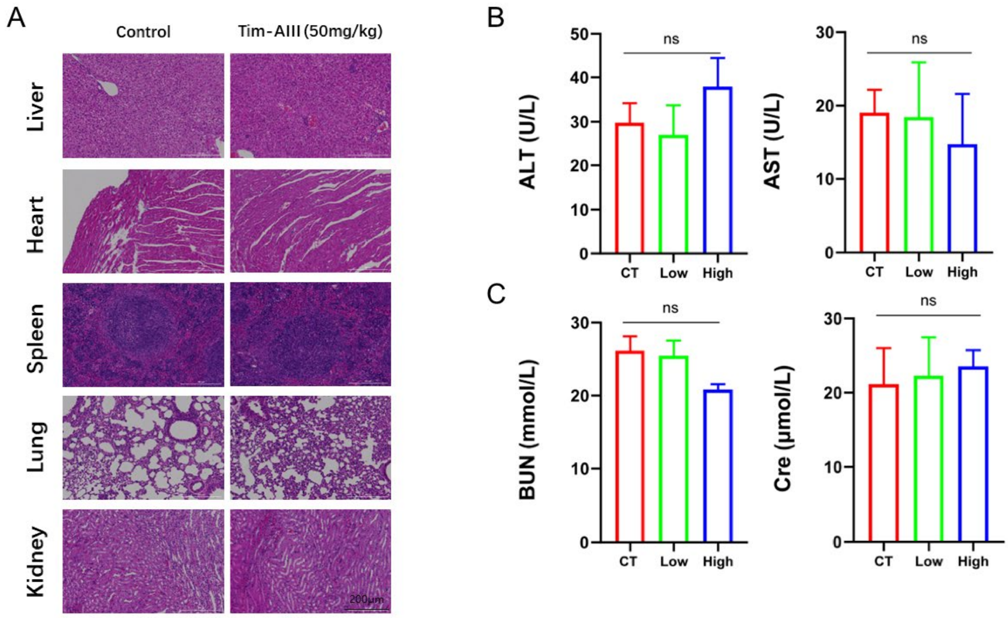

Fig. S4 Tim-AIII is non-toxic to the mice. **A** Hematoxylin and eosin (H&E) staining of liver, heart, spleen, lung and kidney collected from the mice of the high lose treatment and the PBS groups. **B** Serum ALT and AST levels in Tim-AIII administration groups compared with the control group. **C** Serum BUN and Cre levels in Tim-AIII administration groups compared with the control group. Quantitative data were presented as mean ± SD. $^{*}\boldsymbol{p} < 0.05$, $^{**}\boldsymbol{p} < 0.01$, $^{***}\boldsymbol{p} < 0.001$.
